# Stakeholders’ Perception of the Palestinian Health Workforce Accreditation and Regulation System: A Focus on Conceptualization, Influencing Factors and Barriers, and the Way Forward

**DOI:** 10.3390/ijerph19138131

**Published:** 2022-07-02

**Authors:** Shahenaz Najjar, Sali Hafez, Aisha Al Basuoni, Hassan Abu Obaid, Ibrahim Mughnnamin, Hiba Falana, Haya Sultan, Yousef Aljeesh, Mohammed Alkhaldi

**Affiliations:** 1 Faculty of Graduate Studies, Arab American University, Ramallah P.O. Box 240, Palestine; mughnamin@gmail.com (I.M.); hibafalana@yahoo.com (H.F.); haya.sultan11@hotmail.com (H.S.); 2 London School of Hygiene and Tropical Medicine (LSHTM), London WC1H 9SH, UK; sali.hafez@lshtm.ac.uk; 3 Projects Department, Gaza Community Mental Health Programme (GCMHP), Gaza P.O. Box P860, Palestine; aisha_yb@hotmail.com; 4 Indonesian General Hospital, Ministry of Health, Gaza P.O. Box P860, Palestine; hass_aboobaid@hotmail.com; 5 Faculty of Medical Science, Israa University, Gaza P.O. Box P860, Palestine; 6 School of Public Health, Tehran University of Medical Sciences TUMS, P.O. Box 14155-6559, Tehran 14455-6446, Iran; 7 Yatta General Hospital, Ministry of Health, Hebron P.O. Box 785, Palestine; 8 Department of Pharmacy, Nursing and Health Professions, Birzeit University, Ramallah P.O. Box 14, Palestine; 9 Scientific Research and Postgraduate Studies, Faculty of Nursing, Islamic University of Gaza, Gaza P.O. Box 108, Palestine; yjeesh@iugaza.edu.ps; 10 Department of Medicine, McGill University Health Center (MUHC), Montreal, QC H3H 2R9, Canada; 11 Faculty of Medicine, School of Physical & Occupational Therapy, McGill University, Montreal, QC H3G 1Y5, Canada; 12 Department of Environmental Health Sciences, Faculty of Communication, Arts and Sciences, Canadian University Dubai (CUD), Dubai P.O. Box 117781, United Arab Emirates; 13 Health System Impact Fellowship, Canadian Institutes of Health Research (CIHR), Ottawa, ON K1A 0W9, Canada; 14 Faculty of Science, University of Basel, 4003 Basel, Switzerland; 15 Department of Epidemiology and Public Health, Swiss Tropical and Public Health Institute (Swiss TPH), 4123 Allschwil, Switzerland; 16 Research Fairness Initiative Team, Council on Health Research for Development (COHRED), 1211 Geneva, Switzerland

**Keywords:** accreditation and regulations, health workforce, health system, Palestine

## Abstract

The Health Workforce Accreditation and Regulation (HWAR) is a key function of the health system and is the subject of increasing global attention. This study provides an assessment of the factors affecting the Palestinian HWAR system, identifies existing gaps and offers actionable improvement solutions. Data were collected during October and November 2019 in twenty-two semi-structured in-depth interviews conducted with experts, academics, leaders, and policymakers purposely selected from government, academia, and non-governmental organizations. The overall perceptions towards HWAR were inconsistent. The absence of a consolidated HWAR system has led to a lack of communication between actors. Environmental factors also affect HWAR in Palestine. The study highlighted the consensus on addressing further development of HWAR and the subsequent advantages of this enhancement. The current HWAR practices were found to be based on personal initiatives rather than on a systematic evidence-based approach. The need to strengthen law enforcement was raised by numerous participants. Additional challenges were identified, including the lack of knowledge exchange and salary adjustments. HWAR in Palestine needs to be strengthened on the national, institutional, and individual levels through clear and standardized operating processes. All relevant stakeholders should work together through an integrated national accreditation and regulation system.

## 1. Introduction

Health systems (HS) are characterized by a relationship between those who need services and those who are skilled and educated in the delivery of healthcare [1]. The health workforce (HW) is comprised of all persons involved in enhancing health and is an essential block of any functioning HS, without which clinical and public health services cannot be delivered to the population. Human resources are considered one of the six fundamental building blocks in any HS [2]. There is an increased focus on strengthening and developing HW to improve health outcomes, achieve a sustainable and resilient workforce, and attain the global target of Universal Health Coverage (UHC) while addressing existing and emerging health challenges [3].

Health Workforce Accreditation and Regulation (HWAR) is a key pillar of the HS architecture to provide high quality and safe care. Healthcare professional accreditation can be defined as: the process of formal assessment of an educational program, healthcare institution or system using predefined standards that are carried out by an internal or external body to ensure high-quality training for the HW, and safe and high-quality delivery of care [4]. High-standard healthcare delivery and patient satisfaction has increasingly become a top priority for health institutions worldwide [5]. Quality standards stipulate that care should be safe, effective, patient-centered, timely, efficient, and equitable to those who need health services [6]. In response to this patient safety culture in healthcare, there is an increased global emphasis on the assessment of all health care providers for competence, particularly physicians, nurses, and other direct providers, through certification and licensure to meet a standard of accreditation [7]. One of the fundamental components of HWAR relates to the health workforce in the population health domain. Building a skilled public health workforce that has the interdisciplinary skills, knowledge, and attitudes required by multiple professions is one of the main purposes of HWAR. Any healthcare system should provide services for individual patients. It should also support the development of prevention and primary care programs and improve access to these centers. The purpose of HWAR is to build a competent workforce through high-quality institutional training that produces skilled workers who meet specific standards [8]. Leadership, policy, finance, partnership, education, and a system for human resource management are six framework elements that policy makers, managers, and planners should focus on for efficient HWAR system implementation [9]. HWAR processes should include establishing educational standards, assuring the quality of programs, identification of a code of ethics and scope of practice, a licensure system, and a system to ensure workforce development and suitable disciplinary measures. Medical errors and adverse outcomes are less likely to occur among accredited and regulated health professionals [10]. During the COVID-19 pandemic, HSs were consumed by the need to control the pandemic’s spread. The HW played a major role in the pandemic response, but the situation exposed gaps in knowledge and practice that reinforce the importance of accrediting and regulating front-line HW, including in new educational programs such as infection control and mental health.

Accreditation is a common and well-known strategy for improving and assuring health care standards and patient safety, and it remains a priority for the government, medical associations, health organizations, healthcare managers, and other stakeholders [11]. Accreditation is seen as one of the most important management tools for the evaluation of care. It can indicate levels of experience and expertise in addition to a minimum expected standard for any healthcare professional [12].

Differentiating between the contribution of health services and the HS in the strengthening of HW competencies is an important distinction for achieving and sustaining health improvement goals [13]. While health organizations play an important role in responding to future HW challenges, an integrated governance approach is needed that connects HW policy and systems-based government interventions with innovations on the individual organizational level of service management [14]. Governments and other stakeholders, including private and non-governmental organizations (NGOs), should hold themselves accountable for fully implementing the strategies needed to bolster human resources within the healthcare field. Achieving these objectives will require massive investment by both national governments and development partners to provide support, especially to low-income countries, as well as a more efficient use of resources [14]. There is evidence that disconnecting the workforce from reform policy leads to a range of debilitating effects and the new approach of the workforce policy is integrated and team-based health care [15].

Countries in the Middle East region have comparatively novel experience in adopting HWAR. Lebanon was the first country to develop a national accreditation program in 2002 which functions as a governmental tool to regulate and guarantee the quality of care [16]. In 2005, Saudi Arabia established the Central Board for Accreditation of Healthcare Institutions and Jordan established the Health Care Accreditation Council in 2007 [17,18]. Certification and licensure are other forms used to regulate, improve, empower, and accredit healthcare providers and organizations [19]. The major HW challenges in the region are limited employment capacities, a skill distribution imbalance, low retention in rural and remote areas, geographic imbalances, worker performance and motivation, and inadequate management of emigration of HW [20]. In Palestine, the Ministry of Health (MoH) seeks to enforce efficient strategies by identifying individual and organizational needs and assessing the advantages of new policies and interventions. Palestine still has no agreed national accreditation model in hospitals. HWAR is a shared responsibility of the MoH and health professional bodies which accredit and regulate the HW through certification given to all health professionals who have graduated from a registered institution. Some NGOs and private healthcare institutions in Palestine have obtained accreditation from an international body such as the Joint Commission International (JCI) [21]. 

Regardless of improvements in the health sector in many countries, some still have an inadequate HW. This was clearly identified at the onset of the current COVID-19 pandemic crisis when many countries at all socioeconomic levels suffered from shortages within the HW [22]. The nine low and middle-income countries (LMICs) in the MENA region lack human resource health (HRH) policies, as well as having poor planning, limited capacity of educational and training programs, and inefficient HRH management. The region has the second-lowest HRH density (sub-Saharan Africa has the lowest) among the six administrative regions of the World Health Organization (WHO) [23]. In the Eastern Mediterranean Region, and Palestine specifically, HWAR is still lagging and needs serious measures to strengthen both the national policy and institutional levels.

An examination of HW education reveals that throughout the world, countries are experiencing shortages of healthcare workers and policymakers. Attempts to combat these challenges are being made by developing a range of methods and initiatives to optimize the available workforce and achieve the right mix of personnel needed to provide high-quality care [24].

However, HW development needs to be integrated with HS governance and management, health sector policies and legislation, and service delivery strategies and mechanisms [25].

There are limited data about the efficacy of HWAR. However, the literature indicates that accreditation programs that involve healthcare professional certifications are effective in improving outcomes, quality, and safety of care in the hospitals that utilize the accreditation process [18]. Few studies globally have evaluated the perceptions of different HWAR stakeholders. Additionally, there are no reported studies that discuss the Palestinian HWAR process or that assess the perception of health professionals and academics regarding the process. 

The health system in Palestine is largely fragmented and under-resourced. This has an impact on its capacity to tackle shocks and outbreaks. Many calls urging all actors to invest in and reform the health system structure and pillars based on new creative thinking and innovative approaches have been ignored. The system lacks effective governance, evidence-based policies, financing, knowledge and information sharing, resources and technologies, and coordination between health actors. These elements are imperative to design and establish a responsive and resilient health system in such a unique setting [26]. The health system’s preparedness and resilience remain key challenges that became evident during the recent COVID-19 pandemic when healthcare workers were underprepared and suffered from an inconsistent supply chain, with a severe shortage of PPE [27]. The Palestinian health system has four major healthcare providers: (1) the Palestinian Ministry of Health (PMoH) and specialised medical services such as the Military Medical Services; (2) the United Nations Relief and Works Agency for Palestine Refugees (UNRWA); (3) national and international non-governmental organizations (NGOs); and (4) the private sector [28]. The PMoH plays a double role as the leading health regulatory and service provider in Palestine. The PMoH operates approximately one-third of the secondary and tertiary hospitals (27 hospitals from a total of 87), 61% of the total beds of all hospitals and over 61.3% of all primary healthcare facilities in Palestinian Authority areas [29]. The PMoH addresses the growing health demands by contracting private health service providers, particularly in specialized health services in Palestine or abroad. Alongside the PMoH services, the Palestinian Military Medical Services manage three hospitals. In addition, the NGOs and the private sector own 33.2% and 9.8%, respectively, of the total hospital beds in Palestine. Coordination between the PMoH and health service delivery actors remains limited, particularly in health policy formulation and agenda-setting [30]. Health financing in Palestine consists of three key streams: (1) public financing from government sources; (2) private financing from private insurance and out-of-pocket expenditure; and (3) international humanitarian and development aid. In 2012 Palestine exhibited a substantial increase in health expenditures estimated at 15.6% of the gross domestic product, almost as much as many developed countries [29]. Over the period 2000–2011, the government sector contributed 36% on average of health funding, private households’ out-of-pocket expenditure contributed 39%, and non-profit institutions serving households, including UNRWA, contributed 22%, on average, of total health expenditure [31]. Remarkably high levels of expenditure on curative and hospital care made up 85% of government health expenditure [31]. Additionally, a high level of out-of-pocket expenses, including premiums, account for 36.7% of the total spending on health [31]. Out-of-pocket expenses usually represent a financial burden and are likely to restrict access by many to healthcare services.

An assessment of HWAR from different perspectives provides a comprehensive understanding of HWAR that can contribute to more evidence-informed accreditation and regulation design for training and quality improvement. It does so by defining the most important gaps and factors that may affect the HWAR system. The goal of this qualitative research is to explore and describe the conceptual perceptions, governance policy, technical practices, resources and capacity, gaps, and solutions for HWAR in Palestine. The HWAR system has not been sufficiently addressed at the policy making, practice or research levels. This unique national study is the first research that comprehensively addresses HWAR in Palestine by carrying out the study in the two major geographical areas: the West Bank including Jerusalem and the Gaza Strip, and targeting three main relevant sectors: government, academia, and private organizations and NGOs. Specifically, this paper aims to: (1) assess the level of understanding among health policy-makers, academics and experts regarding the definition and conceptualization of the HWAR concept, and ascertain their perceptions of its goals, importance, functions, and application; (2) assess the perceptions of stakeholders about the factors affecting the HWAR system in Palestine and the associated “disadvantages and advantages” of adopting or dispensing with HWAR; and (3) propose actionable recommendations that strengthen the national HWAR in Palestine.

## 2. Materials and Methods

A qualitative research approach was adopted in this study. An inductive approach based on semi-structured interviews with key informants was used to explore and describe the conceptual perceptions, governance policy, technical practices, resource and capacity, gaps, and solutions for HWAR in Palestine.

Participants were selected using a purposive sampling technique. Participants were key informants i.e., health experts, academics, leaders, and policymakers from three sectors: (1) government (Palestinian MoH and Legislative Council); (2) academia; and (3) NGOs from the Gaza Strip and West Bank. As part of this study, all relevant sectors (government, non-profit, academic, and syndicates) were addressed, namely those involved and running HWAR operations in Palestine. Thus, most perspectives are reflected and represented, including assessment and understanding of the topic with a high level of representation, knowledge saturation, and diversity in understanding and perceptions. Participants met the following criteria: (1) they consented to participate in the study, and (2) they have over five years of experience in the field. After obtaining ethical approval from the Helsinki Committee and administration (IRB no PHRC/HC/656/19), invitations were sent to the selected key informants via e-mail. Twenty-two semi-structured face-to-face interviews were conducted comprising eleven key informants from the West Bank and the same number from the Gaza Strip. The interview duration was between 45 min and 90 min, and all interviews were completed between the start of October to mid-November 2019. 

Purposive sampling was used to identify the relevant study participants engaged in HWAR in health sectors through the use of various sampling strategies, typical and critical cases, snowballing, homogenous, and expert sampling. Pre-defined criteria and sampling strategies led to the formation of a list of 22 participants from different sectors. The study captured the experience and perception of 22 study participants. The sample was appropriate and representative where the academics interconnected with other sectors, mainly the government sector which is the main body, including MoH and its relevant departments, the Legislative Council, and the Palestinian National Health Institute, which regulates, governs, and manages HWAR to capture a deeper understanding of the topic. The sample was also diverse and included other local actors such as syndicates, NGOs, and academic institutions. The main interest was first identifying the reality of HWAR policies and practices directly from the relevant government institutions and considering the other sectors’ perspectives. This approach was based on those used in other international and regional studies. It is a feasible and appropriate approach that first takes into account the government’s perspective as the key driver and regulator of the HWAR field, along with the perspectives of others as the second level of understanding.

The checklist for qualitative research—“Consolidated criteria for reporting qualitative research (COREQ): a 32-item checklist for interviews and focus groups” was used to conduct our interviews and to ensure all relevant methodological and reporting aspects were reported [32]. Two researchers (female and male) conducted these interviews. Both have long experience of over ten years in research and conducting interviews. The interview guide (Appendix A), audio recorders (after taking participants’ consent), and transcription were used to guide and report the interviews. The duration of the interviews was from 40–50 min and all interviews took place in an appropriate arrangement as follows: phone communication with the participants and explanation about the study, initial consent, the sharing of guidelines and questions prior to the interview, and then the conducting and recording of the interview.

Inductive analysis was used to develop a pattern and theory around the topic. The study assumes a weak monitoring and evaluation system of HWAR, and its negative consequences on measuring progress towards the governance of human resources in Palestinian health. The research team used a combination of automated qualitative analysis software (MAXQDA 12, 2017 (VERBI GmbH, Berlin, Germany)) and an Excel program for data management and analysis using thematic analysis. The analysis employed a thematic analysis approach using MAXQDA software. The researchers developed a codebook that aligned with the study questions and domains. The researchers coded the transcripts on MAXQDA, and the frequencies of key codes were examined to extrapolate the emerging trends. MAXQDA enabled researchers to compare code frequencies and extract intersecting codes to allow for in-depth analysis of the emerging themes. The thematic analysis was then used to analyze interview data, and to identify and interpret trends across the collected data. The analysis approach comprised six phases including coding, searching, reviewing, defining themes within the analytic narrative, selecting relevant quotes that best represent the overall data, and compiling results into a discussion including relevant literature. Data analysis was completed using MAXQDA 12 as well as manually by two researchers who conducted separate analyses, then discussed each analysis and contested each interpretation to strengthen the analysis process and maintain the rigor of the study.

Each participant received all relevant information regarding the study and was given the choice to participate. Their acceptance was initially verbal and then documented with a signed informed consent form.

## 3. Results

### 3.1. Sample and Participant Characteristics

The participants were aged 38–70 years; 70% of the participants were male and the remaining were female. The gender participation in our study reflects the health system’s structure and design in terms of the gender representation that already exists. Participants were selected based on their direct role, engagement, and positions that relate to HWAR using a purposive sampling approach. The breakdown of participant characteristics can be seen in Table 1.

The key informant interviewees varied in their specialization, background, and affiliations. Half of the interviewees held doctoral degrees and 70% of the interviewees held post-graduate degrees (either masters or doctoral). One-quarter of the interviewees were researchers or academics, and three interviewees came from UN agencies, the World Bank, other NGOs, syndicates, professional unions or were independent experts. The years of experience of the interviewees ranged from 6–40 years and included experience in human resources, health care, or a relevant field. More than half of the interviewees were affiliated with a governmental institution or the MoH both on the central level and in individual facilities.

### 3.2. Conceptualization of HWAR Definition

Participants did not produce a consistent answer or definition when asked about their definition of HWAR. However, half of participants used the human resources-centered definition of HWAR focused on the health service providers themselves, with some extending the definition to administrative and management staff. “*A group of professionals that provide health services to people*” (government expert 1).

The other participants framed HWAR as the process and mechanisms that regulate, govern and operate the licensing of healthcare workers, with some extending the definition to include laws as well. “*Laws and regulations for licensing, entering into and practicing the medical profession*” (academic expert 1).

It is worth highlighting that the majority of participants from Gaza adopted the first human resource-centered definition, while most participants from the West Bank adopted the second definition.

When asked, the majority of study participants had a negative perception of the current status of HWAR in Palestine. Some participants affirmed the lack of progress monitoring tools and relevant indicators, and the absence of evidence-based decision-making on HWAR. The participants highlighted that healthcare workers educated abroad were accredited outside of the Palestinian context and the harmonization of those health workers’ methods of practice within the Palestinian HS is a major gap. In addition, there are no current regulations for re-accreditation, and this may have an impact on the quality of care. A few participants flagged the absence of continuing education requirements. *“There is no monitoring, we do not know the available capacities, and there is no continuous education. There is no so-called re-accreditation of human resources in health according to specific interests or on scientific grounds. There is no study of the subject according to proven scientific principles”* (academic expert 5).

The few participants that expressed positive views on the status of HWAR in Palestine qualified their statements by adding that further development and support of the system are needed. These positive views were attributed to the perception of strong legal frameworks that govern HWAR, and the diverse pool of HW who graduate from universities and institutions inside Palestine and abroad. *“Good and organized, and needs support and development”* (government expert 5).

The question about the main pillars of HWRA in general received diverse answers. Many participants indicated the regulatory bodies and processes associated with HWAR, primarily the MoH, Palestinian Medical Council, general hospital administration, the Foundation for Accreditation and Quality in Higher Education, and professional unions. *“Professional unions of health professions and the MoH”* (government expert 4).

Some participants pointed instead to the cadre of qualified healthcare workers. A few participants highlighted that the HWAR process is underdeveloped.

### 3.3. Advantages of HWAR and the Disadvantages of Its Absence

The majority of the study participants highlighted the advantages of a well-established HWAR system and mechanism rather than the disadvantages of its absence (Table 2). All study participants iterated that strengthening HWAR would contribute to more effective organization and regulation of health service provision and thus, to a higher quality of care and better health outcomes.

*“The wins are safe services of high quality, and vice versa.”*

(government expert 2)

Some participants highlighted other advantages. For example, HWAR’s positive impact on patient safety, increased trust and satisfaction in the HS, and more justice in access to services. *“Increase the quality of services, patient safety, reduce the waste of resources, and increase patient confidence and satisfaction with the health system”* (government expert 5).

Other participants highlighted the contribution of HWAR in reducing unemployment, enhancing resource management, and controlling fraud and malpractice.

*“There is no loss but rather gains in the quality of services, reducing unemployment, providing services when the citizen needs them, reducing the costs of transfers outside Palestine and the effect on the economy, and better working conditions for employees in the organizational process.”*

(government expert 10)

### 3.4. The Current Status of HWAR

When study participants were asked to describe the current status of HWAR in Palestine, over one-third of the participants described it as random, chaotic and disorganized.

*“I would describe HWAR as random, chaos, frustration, with no communication between institutions.”*

(government expert 10)

Many participants highlighted that it is based on personal initiatives and skillsets rather than on a systematic evidence-based approach.

*“The current HWAR is dependent on individual efforts and not on scientific methods.”*

(government expert 1)

The lack of communication between actors was highlighted, along with the need to structure and organize planning around HWAR. *“Random and based on improvised and personal efforts,”* (academic expert 4).

A few participants described HWAR as organized and fit-for-purpose, particularly considering the limited resources and difficult political conditions in Palestine compared with other countries. Some participants highlighted the need for reviews and updates, along with increased enforcement of implementation. *“Very good to excellent compared to other countries. There is institutionalization, but it needs to be modified,”* (government expert 11).

### 3.5. Factors Affecting HWAR in Palestine

Study participants identified the essential factors affecting HWAR. The following factors were expressed most frequently:

1. Political division and the absence of the Legislative Council;
2. Financial resources and mobilization capacities;
3. Multi-stakeholder communication and coordination between the MoH, unions/syndicates, and academic institutions;
4. The regulating laws, procedures and administrative orders;
5. Governance and HS structure;
6. Research, scientific collaboration with academic institutions, learning exchange, and continuous learning and education.

*“Firstly, we do not have a list of criteria that includes knowledge, practices, skill, attitudes, ethics, knowledge update and research. Secondly, there are no material capabilities. Thirdly, there is no system that protects HW.”*

(academic expert 4)

Participants from Gaza identified the political divide between the two administrative systems in the West Bank and Gaza as a factor influencing HWAR more than West Bank participants. Participants affiliated with the MoH repeatedly identified operational factors like the political divide, financial resources, and laws and regulations, while academic participants suggested more HS and governance factors influencing HWAR. “*Political division and the financial situation*,” (government expert 6).

The focus on governance and HS structure included reward and accountability systems, decision-making processes, digitalization of the HWAR process, monitoring and evaluation criteria, transparency and clear job descriptions, roles, and responsibilities.

### 3.6. Problems and Gaps Associated with HWAR

**Figure 1:**
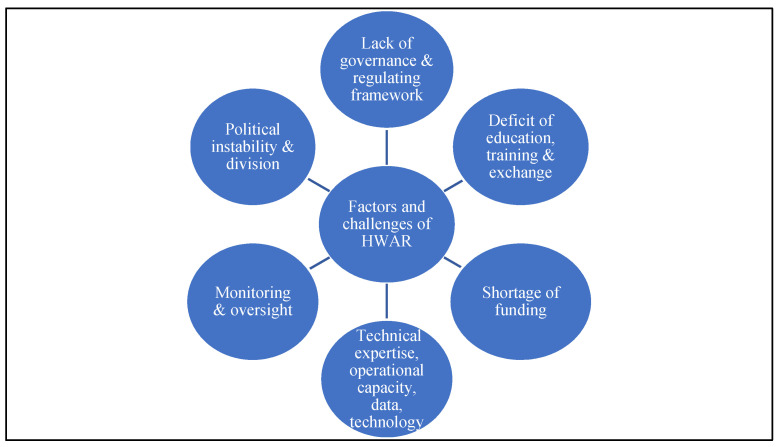
HWAR challenges and gaps.

Asked about the challenges to HWAR in Palestine and their causes, study participants mainly identified political challenges (Figure 1). Half of participants indicated that the ongoing practices of occupation such as closure and siege, and/or political division are the main challenges hindering HWAR progress. Several highlighted that the occupation influences all decision making in Palestine and HWAR is affected by these constraints on potential policy and progress. Participants from Gaza were more likely to identify political instability among the identified challenges. Participants from the West Bank focused more on HWAR governance and system challenges. “*The occupation and its influence on decision-making and control over access to our own resources and the provision of services. Access, human movement, navigation for human resources accountability are challenging. The support of international actors with financial and technical expertise is crucial to us. The legalization of this support has an impact on the MoH. Political division and the lack of national legislation are all key challenges*,” (NGO expert 2).

One-third of participants highlighted that the lack of knowledge exchange, absence of a clear strategy for human resources development, capacity building, and limited scholarship options are among the key challenges faced by the HWAR system. A group of participants also drew attention to the importance of lack of financial resources, salary adjustments, and irregularity among the main challenges. Several participants pointed to human resources challenges beyond capacity building. For example, some explained the absence of clear roles and responsibilities, role division, decision-making processes, and misused competencies and capacities. “*Not putting the right person in the right place*,” (academic expert 5).

Participants also referred to the absence of vision and strategic planning and explained the lack of regulations and instructions on operationalizing these plans. Others highlighted the gap in communication and coordination between the MoH, unions, and academia, as explained earlier in the findings of this study. Several participants highlighted that the current systems lack the organization and clear structure that would strengthen operational capacities.

### 3.7. A Roadmap for an Effective HWAR System

#### 3.7.1. National-Level Recommendations

**Figure 2:**
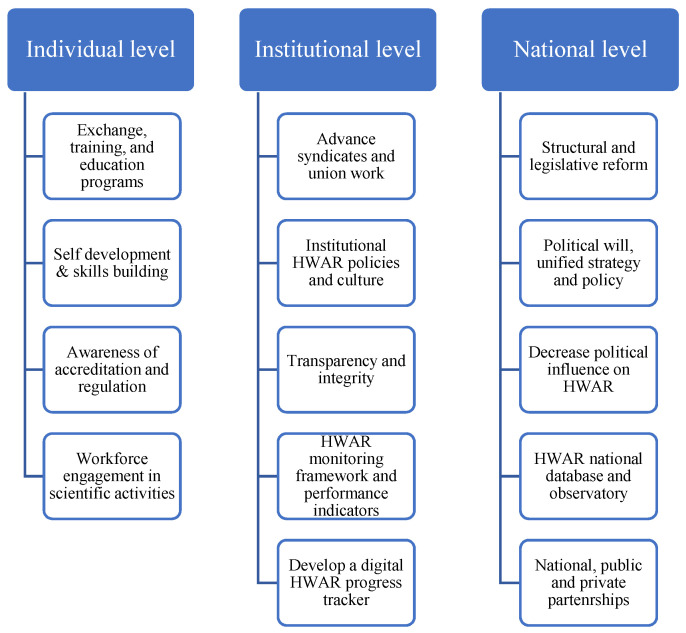
HWAR recommendations on individual, institutional and national levels.

Participants expressed recommendations to address the challenges. They identified key changes that could be made on the national, institutional, and individual levels (Figure 2). At the national level, around two-thirds of participants recommended developing or updating the national strategy and implementation plan of HWAR, including policies, manuals, and guidance. Some participants highlighted that strategy development must engage the MoH in coordination with professional unions and universities. They also pointed out the importance of creating clear processes and standard operating procedures to apply these plans. “*Integration with the Ministry of Higher Education to develop a clear policy for graduates commensurate with the labor market*,” (government expert 3).

Almost half of the participants suggested ending political division, minimizing the impact of political divisions on the healthcare sector, or reconvening the Legislative Council. The majority from Gaza and the MoH pushed for political reform. One-third of the participants recommended developing stronger mechanisms for follow-up, monitoring, evaluation, and data management. This would include evaluating healthcare professionals, monitoring the quality of educational programs, and measuring gaps in human resources in the HS. One participant underlined the need to activate entities charged with monitoring progress and oversight of health resources, like the National Observatory for Human Resources. A quarter of the participants recommended strengthening sector-wide accountability, and transparency policies and measures. Some participants suggested a restructuring of the human resources mechanisms in the HWAR system as central to HWAR governance. Several recommended a review of the hiring process based on competencies, and a few suggested changing the General Council of Employees and establishing a human resources council/ministry. Participants also proposed the establishment of an independent entity concerned solely with HWAR governance. “*Make accreditation and legalization of the HW a top priority for the Ministry. Continuity in work, cooperation and coordination of efforts between the various authorities and ministries. The most important thing is to find a responsible body that can be independent*,” (academic expert 1).

#### 3.7.2. Institutional Recommendations

Around one-third of the study’s participants suggested developing mechanisms to make the implementation of HWAR policies more efficient, including developing guidelines or manuals, standardization of practices, better coordination to link education with practice, and better accreditation procedures. The need to strengthen legal enforcement and implementation of regulations was also raised by participants. A quarter of participants advocated for strengthening the role and capacity of the professional unions and syndicates. One participant suggested developing a new system where professional union membership is granted based on a point system that values the scientific, professional, and personal development of the healthcare provider. “*On the institutional level, we need to activate more union bodies, strengthen the culture of laws and regulations to regulate accreditation and legalize human health resources*,” (United Nations agency expert 1).

Around a quarter of participants recommended developing institutional quality assurance plans and mechanisms. Some expressed the importance of developing efficient systems to track key performance indicators. Others highlighted the need for an entirely new system to monitor and track accreditation, education, re-licensing, and certifications in a digital form. Several participants proposed developing a system for continuing education for healthcare providers with a clear strategy and implementation plan. The importance of knowledge exchange, scientific conferences, and scholarships abroad were also highlighted. The link between continuing medical education and certification was recommended by several participants. “*At the institutional level, organizing educational conferences and workshops and linking them to the hours required for re-accreditation*,” (NGO expert 2).

#### 3.7.3. Individual-Level Recommendations

The majority of the study participants recommended promoting self-development and continuing medical education through workshops, conferences, seminars, and training courses. One participant suggested individual workers be encouraged to seek opportunities for scholarships abroad. Better human resource practices on the individual level were proposed as a way to address challenges faced by the current system. One participant raised the importance of iterative feedback on the development of HWAR. Another participant advised that healthcare workers should be more committed to the full scope of their job description. Stronger engagement with the professional unions was brought up by several participants.

## 4. Discussion

This is the first objective study to assess the comprehension and conceptualization, functions, and applications of the HWAR concept in Palestine. Additionally, it presents an assessment of the overall perceptions of factors affecting the HWAR system in Palestine, existing gaps, and offers solutions to strengthen the current processes. The findings of this study open new avenues for improvements in policy and practices by providing actionable insights for policy makers and healthcare professionals to address this topic.

Overall, there was no consistent definition of HWAR. However, most participants either adopted a human resource-centered definition or one based on laws and regulations for licensing. The majority of participants had a negative perception of the current status of HWAR in Palestine based on the insufficient HW, unstructured HWAR mechanisms, absence of clear job descriptions for different healthcare positions, outdated laws and policies, political challenges, lack of organization, and the lack of clear standards.

According to our findings, HWAR needs further development and support. The participants identified the major gaps in HWAR as lack of progress monitoring tools and relevant indicators, the absence of evidence-based decision-making on HWAR, and the lack of current regulations for re-accreditation or continuing education requirements. Each of these factors has the potential to influence the quality of care and is consistent with the existing literature that addresses HWAR gaps [8].

Regarding the repercussions of the presence or absence of a strong HWAR system, participants indicated advantages to the workforce, health care, population, institution, and system levels. All participants highlighted that strengthening HWAR contributes to better health outcomes, increases the quality of services and patient safety, reduces unemployment, enhances resource management, controls fraud and malpractice, and increases overall patient satisfaction and safety with the HS, alongside increased justice in access to healthcare services. This is consistent with regional and international studies [8,10,15].

Perceptions of the status of HWAR, of the challenges faced, and of the solutions needed were influenced by the geographic region or area of expertise of the participant. For example, different perceptions of HWAR were noted between academic experts who widely reported negative views and described the process as random and based on improvised and personal efforts, versus government experts who reported positive views and described the system as acceptable in comparison with other countries, albeit requiring some institutional modifications. This misalignment regarding the current status of HWAR has not been discussed in any previous studies. The findings did not explicitly show geographical discrepancies but the biggest differences were in three major trends: (1) the political divide, occupation and violence which participants from Gaza highlighted more than participants from West Bank; (2) The impact of the occupation on health service delivery, workforce, learning, study abroad and knowledge exchange was evident more in Gaza; (3) Gaza participants identified challenges regarding the enforcement of legal frameworks and system clarity to a greater extent. Restrictions on resources were also more apparent in Gaza. Otherwise, perceptions, the legal framework, enforcement, and challenges were pretty much the same.

Both academic and governmental experts highlighted several factors that affect the accreditation process and quality of health care. The participants described individual, professional, and political factors such as the political division and absence of the Legislative Council, financial resources and mobilization capacities, multi-stakeholder communication, coordination between the MoH, unions/syndicates and academic institutions, regulatory laws, procedures and administrative orders, governance and health system structure, research, scientific collaboration with academic institutions, knowledge exchange and continuous learning and education. Some of these factors mirror the findings of studies from other countries [25,33,34]. A qualitative study from Pakistan identified many factors affecting the accreditation and regulation process. Two factors identified were: (1) incentives for the healthcare workforce such as a basic salary and working conditions in terms of training, job description, and supervision; and (2) the influence of political conditions, continuity at the top level of the health system, the institutionalization of changes, and the link between information, communication, and sustainability.

Regarding HWAR challenges, the participants outlined some political issues like the Occupation, closures, sieges, or political divisions. These issues were cited in particular by participants from the Gaza Strip whereas HWAR governance and system challenges were highlighted more by the West Bank participants. Other challenges that were clearly identified were the lack of knowledge exchange; absence of a clear strategy for human resources development, capacity building, and limited educational opportunities; the importance of financial resources; salary promotions; unclear roles and responsibilities; lack of evidenced-based decision-making; HW competencies; regulations and guidelines on operationalizing these visions and plans; and gaps in communication and coordination between the MoH, unions, and academia.

These findings are similar to previous studies such as that conducted in Kuwait in 2021 that reported different challenges that burden the accreditation and regulation progress, including staff-related challenges (e.g., high workload, accreditation requirements) and organizational system challenges (e.g., poor teamwork, infrastructure problems). Another study in Lebanon in 2014 identified limited financial resources, staff shortages, resistance to accreditation because it was seen as a vague and new concept, heavy workloads, and poor infrastructure as challenging factors [16,35].

Participants recommended the development of a process of accreditation that functions on three levels: national, institutional, and individual, to bridge existing gaps. On a national level, participants suggested integration with the Ministry of Higher Education to develop a clear policy for graduates commensurate with the labor market. The experts also asserted that accreditation and legal regulation of the HW should be a top priority for the MoH, especially in designing and implementing the current and future pandemic emergency and recovery strategies. Continuity in work and the coordination of efforts between the relevant authorities and ministries were also seen as fundamental to an improved HWAR system. However, the most important recommendation from the participants was to establish a responsible and independent body in charge of accreditation and upholding standards. At the institutional level, recommendations focused on activating more union bodies, strengthening the authority of accreditation laws and regulations, and organizing educational conferences and workshops. Finally, at the individual level, participants recommended promoting self-development and continuing medical education, promoting better human resource practices, utilizing iterative feedback on the development of HWAR that includes both workers and policy makers, and engaging with professional unions with clear job descriptions.

Recently, the Palestinian HS has placed increased priority on patient safety issues and quality assurance. Yet in dealing with the COVID-19 pandemic, the HS has often fallen short. Potential opportunities exist to improve the healthcare system through improving HWAR. The collaboration of multiple stakeholders in the healthcare system, including practitioners, administrators, and health authorities, is essential to make the changes necessary to achieve a functioning HWAR system. Future research is needed to assess the impact of HWAR on patient safety and the quality of care provided. Other research can evaluate the effect of HWAR on patient satisfaction with healthcare services. The study findings remind HS policymakers and experts about the importance of investment in strengthening HWAR, which is a significant benefit either in emergency or non-emergency situations. An effective HWAR will lead to further development in HS preparedness, through the harmonization of sectors and providers, in capacity and resources, and in the efficiency of governance and evidence-based decisions taken in response to the COVID-19 pandemic or future outbreaks and future recovery plans [16].

This study demonstrates multiple strengths as the first study to examine in a structured manner the perception of HWAR in Palestine and the key barriers to its development. The study provides a comprehensive representation of both the West Bank and Gaza. This allowed for the extrapolation of differences in HWAR conceptualization and barriers across multiple geographical locations. As an exploratory study, it offers a window of opportunity for future research in this area. The study sought to collate information as a first attempt to encourage systematic, advanced, and specialized studies on the topic of human resources. The lack of information and data on HWAR is a considerable limitation. Further research is required, using diverse methods, on the effectiveness of existing HWAR mechanisms and processes, particularly the processes of the ministries of health, education, and syndicates. Future research should examine HWAR practices and processes based on indicators and outcomes across different decentralized administrative units and departments, and across stakeholders in the Palestinian health system. Similar studies can also have more participants from policy, managerial, and clinical and professional levels.

Despite its many strengths, the study has several limitations. The limited literature compromises the robustness of the literature and desk review on HWAR within Palestine. Limited quantitative data in this area restricted the triangulation of analysis. Finally, the possible biases of key stakeholders and their affiliations may have over-represented key barriers.

## 5. Conclusions

The HWAR system in Palestine is underdeveloped and disorganized. The study proposes strategic directions and opportunities to strengthen HWAR. The challenges identified in this study were classified into two groups: (1) system-related such as lack of a consolidated HWAR system, communication, monitoring tools, mechanisms, and indicators, and evidence-based decision-making concerning HWAR; (2) environmental factors e.g., the occupation, closure, sieges, or political–administrative divisions that affect HWAR in Palestine. It is acknowledged that HWAR is a national priority and there is a strong legal framework that governs HWAR in Palestine, with the existence of a diverse pool of skilled healthcare workers. It is emphasized that a well-established HWAR system would increase the quality of services, patient safety, reduce the waste of resources, and increase patient confidence in and satisfaction with the HS. Current HWAR practices are personal initiatives rather than systematic and evidence based. HWAR needs to be strengthened nationally by developing a specific strategy that includes policies and manuals. The development of a strategy, led by MoH, should engage all stakeholders in creating clear and standardized operating processes. Institutionally, an effective operational and technical framework is required integrated with the existing legal framework for the HWAR that includes guidelines or manuals, and standards and principles of practice. Individually, it is essential to promote awareness of HWAR, link education with practice, better accreditation, and professional re-practicing procedures, while promoting self-development and continuing medical education. A strong, integrated, and effective HWAR system supports the country and guides the HS to allocate the HW properly to address current and future challenges and outbreaks.

## Figures and Tables

**Table 1:** Participant characteristics.

Variables		*n* (%)
Gender	Female	6 (27.3%)
	Male	16 (72.7%)
Age	Range (Mean, SD *)	38 to 70 years (M = 50.2, SD = 9.9)
Years of experience	Range (Mean, SD)	6 to 40 years (M = 22.5, SD = 9.8)
Highest diploma	Bachelor	5 (22.7%)
	Master	6 (27.3%)
	PhD	11 (50.0%)
Sector	Government	13 (59.1%)
	Universities	6 (27.3%)
	NGOs	3 (13.6%)

* SD = Standard deviation.

**Table 2:** Perceptions of disadvantages and advantages of the HWAR system and process.

Advantages	Disadvantages
**Workforce**	
↑ no. of qualified personnel, positions for qualified personnel, and trusted professionals	Poor professional standards and malpractice
**Healthcare**	
Better safety and quality of care, well-staffed care, improved accessible and affordable care, avoided errors, well-designed and distributed care, reduced medical errors.	Medical errors, unsafe healthcare, poor quality, care inaccessibility, interrupted and disorganized care.
**Population**	
↑ Provider and patient satisfaction, observance of patients’ rights, patient safety	Dissatisfaction among clients and care recipients
**Institution and system**	
Regulated practices, ↓ unemployment, legal responsibility, accountability, individual and institutional protection, reduced referrals abroad, appropriate and healthy work environment, regulated and overseen professions, limits on fraud and forgery, optimal resource allocation and cost-effectiveness, regulated and distributed human resources, provides indicators for assessment of quality of care.	Unorganized regulations and laws, unsystematic practices, interrupted or unprofessional environment in the workplace

## Data Availability

The data presented in this study are available on request from the corresponding author. The data are not publicly available due to privacy or ethical restrictions.

## Appendix A. Assessment of the Health Workforce Accreditation and Regulation in Palestine: A Call for Immediate Policy Actions—Consent Form and KII Guide

**Introduction:**

Thanks for participating in this interview ….warm greetings, interviewer introduces him/herself, and explain briefly the purpose and nature of the interview, as well as its agendas and topics surrounding the HWAR.

**Length of interview:** 45–60 min

**The primary goal of the interview:** To see things the way you see them… more like a conversation with a focus on your understanding, your experience, your opinions, and what you think or feel about the HWAR regulation and accreditation topics covered.

This interview is aimed at investigating the perceptions of the health system’s actors in Palestine to assess the current status of HWAR, to identify the challenges, and to propose useful policy and technical actions for the effectiveness of HWAR. Only national and subnational experts, policymakers, and administrators at public health entities and closely working in HWAR fields are invited to participate.

**Confidentiality and anonymity:** All views, perceptions, and perspectives are only used for research purposes and they will be confidentially saved, managed, and shared anonymously. To maintain this and meet the purpose of our research, the interview will be recorded only in voice, and we kindly ask for your personal permission to do so.

**Verbal Consent:** Two individual consents will be secured, the first initial consent before the interview is conducted when the research team communicates with participants via email or phone to invite and explain to them all study information, and the second consent will be obtained when the interview conducts. Both consents are administered to get the personal approval of participants regarding the participation, data collection, management, and storage, as well as the final publication.

**Mandatory question:**

**Do you agree with the above-mentioned and presented elements related to interview arrangement, confidentiality, consents, participation, and publication?** (Answer Yes or No must be obtained or any other concerns must be reported, then proceed with the KII if the participant agrees)

- Socio-demographic characteristics:

Age:_____ Gender:_______ Level of education: ___________Year of experience: ______ Sector affiliation: ______________Location: ___________ Position: ______________ Contact: __________________________

- Conceptualization

- From your own definition, how do you define the HWAR?
- How do you perceive the HWAR in Palestine?
- When we mention HWAR in Palestine, what comes to your mind first?
- What do we gain from HWAR and what we will lose by giving that up?

- **HWAR governance, policy, and monitoring**

***Governance***

- Is there a clear HWAR system?, describe the governance structure of HWAR in Palestine?
- Who governs the HWAR system (specific bodies), and how do you perceive the roles of these bodies?
- Describe the process and mechanisms of accreditation, licensing, and quality assurance of health resources?

***Policy***

- Based on your knowledge and experience, is there a national policy and institutional guidelines or manual for managing the work of HWAR? Please justify?

**Monitoring**

- Are there monitoring, verification, and evaluation operational plans that include indicators and criteria for the HWAR processes and practices in the Palestinian MOH? Please justify why?

- **Practices**

- Are there specific accreditation and regulation frameworks and competency standards for each health profession?, are they applied properly? why?
- Could you describe the current status of HWAR in Palestine?
- Are the current accreditation and regulation practices actually started with examining education programs and ended to practice in the profession?
- Do think these practices are performed in high-quality, rigorous, and appropriate? why?

- **HWAR resources and capacity**

- Do you think that the current HWAR personnel is qualified, and the financial resources and logistical capacity of HWAR are sufficient? why?

- **Factors and gaps**

- What factors or issues affect the current practices and processes of HWAR in Palestine? list them, please.
- In general, what are the most common challenges that the HWAR system is facing and what are the causes? mention as bullet points, please.

- **Suggestions and solutions**

- Based on your understanding, what you suggest recommendations and developmental actions for a better HWAR in Palestine? At:
  1. National level
  2. Institutional level, and
  3. Individual-level
- What resources and support are needed to implement those interventions or actions?

**Additional comments, is there anything else you would like to share with us or any questions you have for us?**

**The end…**
